# The economic consequences of attention-deficit hyperactivity disorder in the Scottish prison system

**DOI:** 10.1186/s12888-018-1792-x

**Published:** 2018-06-25

**Authors:** Susan Young, Rafael A. González, Moshe Fridman, Paul Hodgkins, Keira Kim, Gisli H. Gudjonsson

**Affiliations:** 1 Psychology Services Limited, PO Box 1735, Croydon, CR97AE UK; 2 Reykjavik University, Reykjavik, Iceland; 3 East London NHS Foundation Trust, London, UK; 4 AMF Consulting Inc, Los Angeles, CA USA; 5 Sage Therapeutics, Cambridge, MA USA; 6 Indedpendent Medical Writer, San Diego, CA USA; 7 Institute of Psychiatry, Psychology and Neuroscience, King’s College London, London, UK

**Keywords:** ADHD, Health-related quality of life, Economic evaluation, Quality-adjusted life years, Prison, Costs

## Abstract

**Background:**

Attention-deficit hyperactivity disorder (ADHD) is highly prevalent amongst prison inmates and the criminal justice system (CJS) likely bears considerable costs for offenders with ADHD. We aimed to examine the relationship between ADHD and health-related quality of life (HRQoL) and quality-adjusted life years (QALY) amongst imprisoned adults; and to estimate the annual expenditure associated with ADHD status in prison.

**Methods:**

An observational study was performed in 2011–2013, at Porterfield Prison, Inverness, United Kingdom (UK). The all male sample included 390 adult prison inmates with capacity to consent and no history of moderate or severe intellectual disability. Participants were interviewed using the Diagnostic Interview for ADHD in Adults 2.0. The Health Utilities Index Mark 3 (HUI3) was used to measure health status, and to calculate attribute specific HRQoL scores and QALY. Health service utilisation was obtained through inspection of medical prison records. Inmates with ADHD were compared with inmates without ADHD.

**Results:**

Inmates with ADHD had significantly lower QALYs, with a clinically significant adjusted difference of 0.13. Psychiatric co-morbidity accounted for the variation of ADHD on the HUI3 emotion domain only. Medical costs for inmates with ADHD were significantly higher; and behaviour-related prison costs were similar to prisoners without ADHD, reflecting a low frequency of recorded critical incidents.

**Conclusions:**

ADHD may directly contribute to adverse health and quality of life through cognitive and executive function deficits, and co-morbid disorders. The extrapolation of conservative cost estimates suggests that the financial burden of medical and behavior-related prison care for inmates with ADHD in the UK is approximately £11.7 million annually. The reported cost estimates are conservative as there is great variability in recorded critical incidents in prisons. In turn, for some prison establishments the prison care costs associated with prisoners with ADHD may be considerably greater.

## Background

Among the general population attention-deficit hyperactivity disorder (ADHD) confers significant financial burden [1, 2] and given the disproportionate prevalence in the prison population, the criminal justice system (CJS) likely bears considerable economic consequences for offenders with ADHD.

ADHD is a childhood onset neurodevelopmental disorder [3] often persisting into adulthood. It is one of the most common mental health disorders in children with recent prevalence estimates ranging between 5.9 and 7.1% [4, 5]. There is recent evidence suggesting late-onset of ADHD, which will require research to better understand its implication [6, 7]. Clinically significant symptoms persist beyond childhood in 65% of cases [8], and may affect as much as 2.8–5.3% of adults worldwide [9, 10]. Its substantial burden of disease is evidenced by an increased likelihood for serious accidents [11], earlier mortality rates [12], substance dependence [13], criminality, incarceration, and false confessions [14]. ADHD confers significant impairment [15], and reduced quality of life [16] to those afflicted by it. It is also highly prevalent amongst prison inmates, with a meta-analytical prevalence estimate of 25.5% [17]. Prison inmates with ADHD are reported to be at significant risk for having increased psychiatric co-morbidity and poorer psychosocial adjustment to the prison environment [18–21].

Health economic evaluations have become an essential part of research and provide evidence supporting health interventions [22]. ADHD is consistently linked with substantially elevated costs and with significant economic burden on education [23] and health [2]. Annual service costs linked with ADHD are reportedly £670 million in the UK [24]. Meanwhile, annual ADHD-related healthcare costs are estimated between $21 to $44 billion in the United States [23]. Furthermore, in the US costs associated with accident claims are more than three times higher in adults with ADHD [1].

Despite the disproportionate representation of ADHD within the prison population, the health-related quality of life (HRQoL) and related costs remains unknown.

In this study we aim to examine the impact of ADHD amongst imprisoned adults. We set out to determine prisoners’: 1) scope and extent of impaired HRQoL utility scores and quality-adjusted life years (QALY) and 2) service use and costs attributable to ADHD.

## Methods

### Participants and sample selection

Following approval from the Scottish Prison Service Research Access and Ethics Committee and in accordance with the Declaration of Helsinki written consent was obtained by prisoners who were recruited by opportunity sampling from Porterfield Prison, Inverness, Scotland, UK, over a period of 18 months in 2011–2013. Participants included 390 adult male prisoners who consented to participate. Those with moderate or severe learning difficulties, lack of fluency in the English language, and severe mental illness (as judged by prison officers) were excluded from participating.

Participants in the study were indirectly compensated. The study group deposited £20 per participant into a Prison Common Good Fund, which was managed by a group of prisoners. The fund was then used to purchase items for the common good of all prisoners to enhance prison life.

Prisoners who indicated interest attended an appointment with the researcher where they were given detailed oral and written information about the study and the consent procedures. After obtaining written consent, researchers administered a comprehensive battery of measures, which took approximately 4 h to complete (usually split across 2 or 3 sessions). The researchers received comprehensive training to administer the measures from the Maudsley Hospital Adult ADHD Service. Further details about the comprehensive battery of measures have been published elsewhere [25].

Data related to medical service use were gathered through inspection of prison medical records and medical costs were calculated based on reference costs reported by the (NHS; see details below). Data related to behavioural disturbance incidents were gathered through inspection of prison records and related costs were calculated based on similar reference costs and were reported as prison costs (see details below).

### Measures

#### Health utilities index mark 3 (HUI3)

The HUI3 is a multi-attribute health status classification system that enables researchers to map levels in the following categories: vision, hearing, speech, ambulation, dexterity, emotion, cognition, and pain; using decision tables and coding algorithms, which can be represented in terms of attribute specific HRQoL scores [26, 27]. HRQoL refers to the value assigned to life span when considering impairments and functional states that may be affected by disease, injury, and treatment [28]. The HUI3 scoring system provides HRQoL utility scores ranging from 0.00 (dead) to 1.00 (perfect health), and meets criteria for calculating QALY [26]. Prisoners’ were asked to answer HUI3 questions based upon their health status in the 4 weeks prior to the interview. The HUI3 composite score was used to calculate QALY and was extrapolated to 1 year to represent the study health evaluation time frame, as previously applied on cost-effectiveness studies [29]. Estimating beyond this time frame would have introduced a very high degree of uncertainty in estimates.

#### ADHD diagnosis

All participants underwent a comprehensive evaluation for ADHD and were interviewed using the Diagnostic Interview for ADHD in Adults 2.0 (DIVA-2) [30]. The DIVA-2 is a validated semi-structured clinical interview used to diagnose ADHD in adults based on the 5th edition of Diagnostic and Statistical Manual of Mental Disorders (DSM-5) criteria [3]; and has been used in clinical [31] and law enforcement settings [32]. Questions addressed their current and childhood (ages 5 to 12) presentation of ADHD symptoms and scope of impairment.

Participants were also questioned whether they were previously diagnosed or treated for ADHD or any other psychiatric illness.

#### Brief symptom inventory (BSI)

The Brief Symptom Inventory (BSI) is a brief psychological self-report scale [33]. The BSI has 9 subscales (Somatization, Obsession-compulsion, Interpersonal sensitivity, Depression, Anxiety, Hostility, Phobic anxiety, Paranoid ideation and Psychoticism), and 3 composite measures (Global Severity Index, Positive Symptom Distress Index, and Positive Symptom Total). We used the BSI depression and anxiety measures as covariates in our health evaluation models because they represent common mental health conditions.

#### Medical service use and costs

Detailed medical service utilization history was obtained through inspection of participants prison medical records. Data from prisoners’ medical charts (covering the 3 months prior to the appointment with the researcher) were abstracted, verified, and entered into a database for analyses. The authors chose to include 3 months of service for practical reasons; and additionally thought this time period fairly represented the medical service use of all prisoners given the variance in prison stays. Data included details from appointments with a general practitioner, physical health nurse, mental health nurse, addiction services nurses, or any other type of nurse, psychiatrist, psychologist, podiatrist, oral health practictioner, or any other type of health related visit such as Well-man clinic or other health clinics, and hospital outpatient visit. Medical costs for these appointments were calculated according to reference costs reported by the NHS Trust [34]. Medication costs were not explicitly collected in the study.

#### Prison service use and costs

Prisoners’ behavioural disturbance incidents were obtained from prison records. Reports of non-attendance to prison activities, being under observation, number of adjudications, and critical incidents were collected and used to calculate the related prison costs. Prison costs were calculated based upon reference costs from the UK Ministry of Justice and HM Prison Service [35], Social Research Unit, Dartington [36], and from direct communication with Scottish Prison Service management.

All reported costs were in Pounds Sterling (£) for the years 2012–2015, and adjusted using the Consumer Price Index (CPI, 2016).

### Analytical strategy

Frequencies were reported for all categorical variables, and means with their standard deviation for continuous variables. The median and inter-quartile range was used for all cost related values.

Because of the HUI3 utility scores’ interval properties, we used t-tests for unadjusted analyses. To estimate the association between ADHD and HUI3 single attribute and composite utility scores, Type I *Tobit* models were used in favour of traditional ordered logistic regression models. A *Tobit* model is designed to estimate linear relationships between variables when there are ceiling or flooring effects on the outcome [37]. Ignoring the censoring and fitting regression models estimated using OLS would have been systematically biased toward the null hypothesis, whereby type II error is increased. HUI3 single attribute and composite utility scores with the value of one are considered censored.

Considering the highly skewed nature of cost variables we used generalised linear models (GLM) with a gamma distribution and log-link function. This way the natural log is modeled and then the predicted margins are calculated in order to obtain the cost differential for those with ADHD [38]. All cost models were adjusted only for age. HUI3 includes an emotion domain that may be sensitive to coexisting disorders (in addition to ADHD). Therefore, models for all HUI3 variables were further adjusted for co-morbid anxiety and depression standardized symptom scores.

We established a significance level at *p* ≤ 0.05 for all statistical tests. All analyses were performed using Stata version 13 (StataCorp) [39].

## Results

### Descriptive statistics

The all male sample was essentially Caucasian British (99.0%) with an average age of 30.3 years (sd 8.3). Prisoners with ADHD had a significantly lower mean age than those without ADHD (28.2 years (sd 7.5) vs. 31.0 years (sd 8.5,) *p* < 0.01). 18.8% (18/96) prisoners with ADHD reported a prior diagnosis of ADHD and 15.6% (15/96) reported having ever received pharmacological treatment for ADHD.

Out of the total sample of 390 participants, 81 (20.8%) required assistance with reading the questionnaires. For those diagnosed with ADHD, 31/96 (32.3%) required assistance in contrast to 50/294 (17.0%) of the other participants. This difference is significant (Chi^2^ (*df* 1) = 10.2, *p* = 0.001; Odds Ratio = 2.3, Confidence Interval 1.3–3.9).

### Health status

Table 1 includes the mean and distribution of all HUI3 specific attributes and composite HRQoL utility scores for all inmates. Prisoners’ variability noticeably increased in scores for emotion, cognition, pain, and HRQoL.

**Table 1:** Distribution of HUI3 Specific Attribute Utility Scores for all Inmates

HUI3 Attributes, *N* = 390	n	Mean score	Standard deviation
Vision	385	0.989	(0.024)
Hearing	387	0.989	(0.061)
Speech	390	0.966	(0.073)
Ambulation	389	0.991	(0.072)
Dexterity	390	0.985	(0.078)
Emotion	370	0.787	(0.274)
Cognition	390	0.837	(0.237)
Pain	390	0.854	(0.278)
HRQoL	362	0.647	(0.307)

Notes: HUI3 data covered the 4 weeks prior to interview

Cost per unit is defined as cost per inmate for each visit. Median medical costs were calculated from data collected from medical records covering the 3 months prior to interview

^a^Includes Hepatitis C, Harm reduction, and Smoking cessation nursing staff

^b^Includes any other recorded health visits, e.g. Well-man clinic

^c^Includes all missed prison therapeutic and other activities per inmate

^d^Calculated by time spent and number of prison staff involved (based on average hourly pay rate)

^e^Calculated by time spent and number of prison staff involved in any adjudication process (estimated 15 min. Per process)

**Fig. 1:**
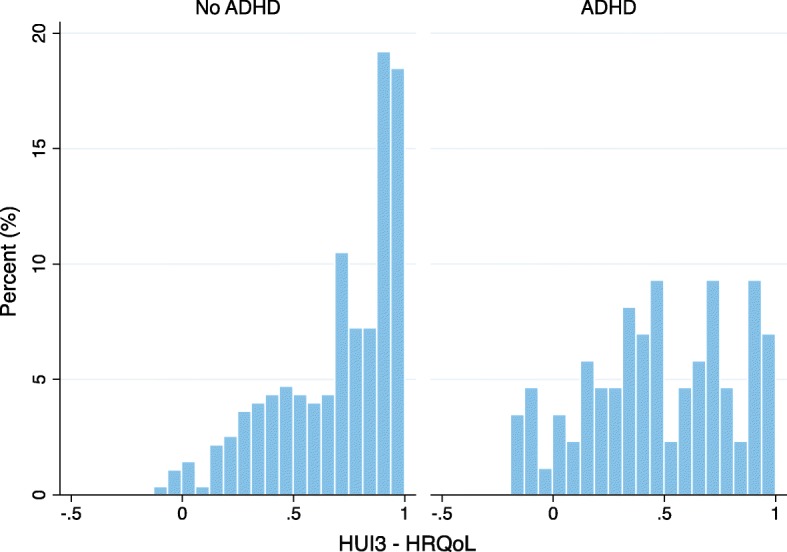
Distribution of HUI3 Composite HRQoL scores by ADHD group: A greater proportion of inmates without ADHD have HUI composite HRQoL scores above 0.7. There is a greater amount of variability in the HUI composite HRQoL scores among inmates with ADHD

Independent sample t-tests were estimated for all utility scores and HRQoL (Table 2) comparing prisoners with ADHD with prisoners without ADHD. Inmates with ADHD had significantly lower scores in the following categories: speech (*p* < 0.05), ambulation (*p* < 0.01), emotion (*p* < 0.001), cognition (*p* < 0.001), pain (*p* < 0.05), and HRQoL composite (*p* < 0.001). Figure 1 shows the distribution of HRQoL utility scores comparing prisoners without ADHD with those with ADHD.

**Table 2:** HUI3 Attribute Utility Scores by ADHD Group (*n* = 96 ADHD, *n* = 294 non-ADHD)

HUI3 Attributes	No ADHD Mean score	ADHD Mean score	*t*	Effect size (*d*)	*p*
Vision	0.991	0.986	1.839	0.218	*0.067*
Hearing	0.992	0.979	1.938	0.229	*0.053*
Speech	0.971	0.952	2.20	0.259	*0.028*
Ambulation	0.997	0.974	2.71	0.319	*0.007*
Dexterity	0.984	0.986	−0.113	−0.013	*0.910*
Emotion	0.817	0.692	3.83	0.466	*< 0.001*
Cognition	0.879	0.709	6.41	0.753	*< 0.001*
Pain	0.874	0.792	2.52	0.296	*0.012*
HUI3 Composite HRQoL	0.699	0.477	6.15	0.759	*< 0.001*

Table 3 demonstrates that even after adjustment for age, anxiety, depression, and/or without correction for missing values, that censored *Tobit* models were significant in each adjusted model for vision, ambulation, emotion, cognition, and QALY. The inclusion of anxiety and depressive disorders in models 2 and 3, attenuated the associations with emotion, hearing, and pain attributes. Attenuation of the association with hearing and pain should be interpreted with caution, as their unadjusted effect sizes were small. Models 2 and 3 show that prisoners with ADHD have a significant difference in QALY of 0.13 and 0.10, compared to those without ADHD.

**Table 3:** Adjusted Regression Models for HUI3 Attribute Utility Scores for Inmates with ADHD Compared with Inmates without ADHD; (*n* = 96 ADHD, *n* = 294 non-ADHD)

	Model 1	Model 2	Model 3
HUI3 attributes	Beta coef. (se)	Beta coef. (se)	Beta coef. (se)
Vision	−0.04 (0.01)**	− 0.04 (0.01)**	− 0.04 (0.01)**
Hearing	− 0.48 (0.22)*	− 0.30 (0.20)	–
Speech	−0.08 (0.04)	−0.05 (0.05)	− 0.05 (0.05)
Ambulation	−0.51 (0.23)*	− 0.54 (0.25)*	− 0.54 (0.25)*
Dexterity	− 0.06 (0.11)	− 0.03 (0.12)	− 0.03 (0.12)
Emotion	−0.19 (0.04)***	− 0.05 (0.04)	−0.06 (0.04)
Cognition	−0.29 (0.05)***	− 0.20 (0.05)***	− 0.20 (0.05)***
Pain	− 0.15 (0.07)*	− 0.02 (0.07)	−0.02 (0.07)
QALY derived by HUI3^a^	−0.25 (0.04)***	− 0.13(0.04)***	− 0.10 (0.04)*

Note. The first two columns refer to Tobit models using data that was corrected for ‘don’t know’ answers. The third column includes the sensitivity analysis, in which we fitted a similar Tobit model but using only available data without accounting for ‘don’t know’ answers

^a^All tobit models to account for censoring at the upper level of the outcome QALY

Model 1 is adjusted for age

Model 2 is adjusted for age + BSI anxiety + BSI depression

Model 3 is adjusted for age + BSI anxiety + BSI depression and is a sensitivity analysis of the sample without correction for ‘don’t know’ answers

*p < 0.05

**p < 0.01

***p < 0.001

25% of all participants had missing values by endorsing ‘Don’t Know’ on several questions of the HUI3. Patterns of missing values were analysed and the most plausible values were imputed using a technique developed by Naeim and colleagues specifically for HUI-3 scores [40].

All 41 questions of the HUI-3 instrument allow respondents to answer ‘Don’t Know’. Because there are no instructions in the instrument manual for how to manage or score these answers, the ‘Don’t Know’ category interferes with the scoring leading to substantial amounts of missing data. Common methods for imputing data in this scenario may not be as effective or can even be misleading, given that answers to other questions within the same domain (e.g. vision) often help identify a sole correct answer to those marked as ‘Don’t Know’. The well cited imputation method by Naeim et al. [40] advises inspecting each possible change in attribute score for every answer to the ‘Don’t Know’ missing value, then selecting the most plausible value accordingly.

Furthermore, we performed a sensitivity analysis based only on those with complete HUI3 data to examine any differences in estimates before and after using the inspection and deduction method to account for ‘Don’t Know’ answers.

### Service utilization and costs

Table 4 includes all cost model inputs for the total median associated medical and prison costs for all inmates.

**Table 4:** Three-month Medical and Prison Costs for All 390 Inmates

	Cost(£)/unit	Median cost over 3 months (£)	Inter-quartile range (£)
Medical costs per inmate			
General practitioner	121	242	242
Physical health nurse	74.3	74.3	74.3
Mental health nurse	78.5	78.5	78.5
Addiction nurse	78.5	78.5	78.5
Nursing other^a^	74.3	0	0
Psychiatrist	148.6	0	0
Psychologist	142.2	0	0
Podiatrist	34	0	0
Oral health	87	0	0
Other health visit^b^	87	0	0
Hospital outpatient	125	0	0
Prison costs per inmate			
Activities non-attendance^c^	38.2	0	76.4
Observation level^d^	13.3	0	0
Adjudications^e^	21.4	0	0
Critical incidents^d^	13.3	0	0
Overall medical costs	–	464.9	488.3
Overall prison costs	–	34.7	76.4
Overall medical and prison costs	–	503.1	532.6

Notes: Cost per unit is defined as cost per inmate for each visit. Median medical costs were calculated from data collected from medical records covering the 3 months prior to interview

^a^Includes Hepatitis C, Harm reduction, and Smoking cessation nursing staff

^b^Includes any other recorded health visits, e.g. Well-man clinic

^c^Includes all missed prison therapeutic and other activities per inmate

^d^Calculated by time spent and number of prison staff involved (based on average hourly pay rate)

^e^Calculated by time spent and number of prison staff involved in any adjudication process (estimated 15 min. Per process)

Table 5 shows that in terms of medical service utilisation, prisoners with ADHD visited significantly more general practitioners (*p* < 0.05), physical health nurses (*p* < 0.05), and mental health nurses (*p* < 0.01) in the three-month period assessed. No significant associations were observed for any other health services.

**Table 5:** Three-month Medical Service Utilisation for Inmates with ADHD (Compared with Inmates without ADHD; *n* = 96 ADHD, *n* = 294 non-ADHD)

Medical service use	beta coefficient (se)	IRR (95%CI)	*p*
General practitioner	0.19 (0.09)	1.21 (1.00, 1.45)	0.04
Physical health nurse	0.22 (0.10)	1.25 (1.03, 1.50)	0.02
Mental health nurse^a^	0.61 (0.22)	1.84 (1.19, 2.85)	0.01
Addiction nurse	−0.11 (0.21)	0.90 (0.59, 1.35)	0.60
Other nurse^b^	− 0.18 (0.54)	0.83 (0.29, 2.40)	0.74
Psychiatrist and Psychologist	−0.12 (0.80)	0.89 (0.19, 4.27)	0.89
Oral health	0.93 (1.46)	2.54 (0.14, 44.74)	0.53
Other health related visit^c^	− 1.85 (0.99)	0.16 (0.02, 1.09)	0.06
Hospital outpatient	0.56 (0.54)	1.76 (0.61, 5.04)	0.30

Note: Visits to Podiatrist excluded for *n* < 10

^a^Includes general mental health and psychiatric nursing staff

^b^Includes Hepatitis C, Harm reduction, and Smoking cessation nursing staff

^c^Includes any other recorded health visits, e.g. Well-man clinic

Table 6 shows that age adjusted medical costs were significantly greater among inmates with ADHD (*p* < 0.05), but prison costs were not. Cost items were assessed based on a 3 month window, then calculated for 1 year assuming similar patterns of health service utilisation and behaviour in prison. Total medical and prison costs for inmates with ADHD were £ 590 more per year than for inmates without ADHD.

**Table 6:** Average Costs Model Results and Estimated Marginal Predictions for Inmates with ADHD (Compared with Inmates without ADHD; *n* = 96 ADHD, *n* = 294 non-ADHD)

	3 month beta coefficient	3 month Predicted margin	1 year Predicted margin
Medical Costs	0.25*	£ 135.9	£ 543.6
Prison Costs	0.25	£ 11.4	£ 45.6
Total Costs	0.24**	£ 147.5	£ 590

Note: Findings from Generalised Linear Model using gamma error distribution and log link function, adjusted for age

*p < 0.05

**p < 0.01

## Discussion

### HUI3 health attributes and QALY

To the best of our knowledge, our study is the first addressing ADHD health status using HUI3 amongst prison inmates. Previous studies documented the relationship between symptom severity and poorer HRQoL, including somatic symptoms [41], whereas a UK cross-sectional study reported that across most health domains, children and adolescents with ADHD had poorer scores when compared with samples of children with diabetes, and a healthy comparison group [42]. These studies highlight the extent that ADHD has on the health impact on affected individuals.

We analysed the role of ADHD on QALY based on a one-year horizon. Notably, the proportion of inmates with a HRQoL over 0.90 (healthy state) was vastly superior amongst those without ADHD. The final adjusted model that accounted for psychiatric co-morbidity produced a 0.13 difference in QALY, four-fold above the 0.03 clinically relevant threshold estimated by the instrument developers [26]. QALY based on inmates’ one-year health utility scores for those with ADHD were significantly lower than those without ADHD. Poorer specific health attribute scores on vision and mobility indicate that inmates with ADHD have significantly compromised health states that go beyond of those more usually expected (such as emotion and cognition) from the disorder. Furthermore, health utilities models adjusted for psychiatric co-morbidity accounted for the variation of ADHD on emotion aspects, but not on the cognition attribute, providing an important insight regarding the contributing factors to impairment amongst inmates with ADHD.

The significantly poorer vision score among the ADHD group may relate to their reading difficulties. In the present study, those diagnosed with ADHD were over two times more likely to require assistance with reading the questionnaires than the other participants. With respect to mobility, the finding that prisoners with ADHD have significantly poorer ambulation problems may reflect that prisoners with ADHD suffer more injuries that hinder their mobility compared with non-ADHD prisoners. Data obtained from the Danish registry reported that the morbidity rate is nearly three times higher if you have ADHD, and that 77.7% of unnatural deaths were accounted for by accidental injury [12]. Additionally, given the higher rates of aggression and violence in the ADHD population [43], there may be mobility problems arising from assault.

ADHD is frequently reported to be associated with a substantial reduction in the quality of life of children [44] and with increased chronic health problems in adults [45]. Study results indicate that ADHD impacts HRQoL with severe effects in emotional and social domains, and at least moderate effects in physical domains [46, 47]. Adult inmates in our sample had an unadjusted HRQoL of less than 0.60. It is likely that undiagnosed and untreated ADHD has a cumulative effect and increases the risk for further health impairments, especially among imprisoned adults with coexisting mental health and social problems.

There is evidence to suggest that poor HRQoL in individuals with ADHD may be driven by the existence of co-morbid conditions [48]. In our study, although co-morbidity played a role in the impact of ADHD on HRQoL, the association is not entirely explained by coexisting psychiatric symptoms of anxiety and depression. Moreover, there was no attenuation on the association with the cognitive attribute of the HUI3 on adjusted models, suggesting a domain-specific link. Cognitive dysfunction in the form of difficulties allocating attentional resources [49], response inhibition, and management of reward are hallmarks of the ADHD phenotypic expression. These results denote different paths through which ADHD may impact adverse health and quality of life, directly through cognitive deficits and via co-morbid disorders. We therefore provide evidence of domain-specific and shared contributions to impaired HRQoL in ADHD.

### Service use and costs

Health economic studies on the general population report that ADHD (including symptoms of hyperactivity) is associated with significant economic burden [1, 2]; however, studies focusing exclusively on the economic impact of ADHD on adult prisoners were not identified.

A US study of disability claims reported that patients with ADHD had 2.6 more medical claims than those without ADHD and that ADHD imposed a significant financial burden [1]. A recent prospective UK study reported that preschoolers with high levels of hyperactivity had a 17-fold increase in overall costs compared with non-hyperactive controls; costs were mainly driven by mental health, educational, social, and criminal justice system service use [2]. A Danish study reported that the direct medical costs of ADHD patients were relatively high, whereof mental care and inpatient hospitalizations accounted for approximately 60% of the costs and medication use accounted for 13% [50]. Results of one study demonstrated that public costs (due to mental health, school services, and the juvenile justice system) are more than double for youth with ADHD compared with those without ADHD [51].

Hospital inpatient stays are a significant driver of costs attributable to ADHD. A retrospective analysis during a 9 year period reported that median hospital inpatient, hospital outpatient, or ED admission costs for individuals with ADHD were more than double for those without ADHD [52]. Pharmacotherapy costs are also a large part of medical costs attributable to ADHD. Medication costs were reported to account for about 13–38% of total costs [1, 2, 24, 50, 52, 53]. Psychological therapy (individual or group modalities) is often another common important driver of costs, which was essentially not utilised by the participants of our study.

Our total estimated annual cost of £590 per inmate with ADHD demonstrates that the costs attributable to ADHD are relatively high. But because our estimate did not include costs for hospital stays, medication, and/or psychological treatment, the total cost estimate therefore represents a conservative figure.

In our study, costs associated with ADHD were driven by increased medical service use and not by behavioural disturbance incidents. This may indicate that costs related to behavioural incidents were more generally distributed across the prison sample and due to many other factors besides having a diagnosis of ADHD. Service utilisation patterns were restricted to general medical and nursing services. Low endorsement of engagement with these and other services may have been a true reflection of the patterns of use in our sample, or of the Scottish prison system at large. As many resources were not used, costs remained lower compared with other studies mentioned.

Because the present study found there were significantly greater medical costs but not behaviour-related prison costs, the cost implication seems to be largely for the NHS. While the assignment of prisoner medical costs based on NHS reimbursements may not perfectly represent prisoners medical costs (possibly over- or under-estimated), it helped to estimate and interpret costs using standard more widely used and familiar terms. There may be, however, variability in the recording of critical incident data, which will be a fundamental driver of prison costs, leading to increased number of seclusions, adjudications, injury costs and potentially staff sickness. In a previous study conducted in a large prison in Aberdeen there were highly significant differences found in aggressive critical incidents between an ADHD and a non-ADHD group [43]. Hence for some prison establishments, costs to the prison service may be considerably higher.

### Limitations

A key strength of the study is its large sample size and a methodology in which every participant was clinically diagnosed using the DIVA-2. Nonetheless, there are several limitations.

Because of missing data, some bias may be present in our analyses of HUI3 specific attribute scores. However, our models accounted for missing data using a well established and oft cited method and the sensitivity analysis on adjusted models allowed us to have confidence in our methods.

Ethnic minority groups and females did not have representation in this sample, therefore, it remains unclear whether these findings may be fully applicable and generalized to the entire prison population.

ADHD diagnosis was based on self-reported information and we did not include informant (e.g. familial) reports. Recall bias is unaccounted for and may have been a factor in symptom measures and service use. Nevertheless, any bias related to under-reporting was presumed to have similar effects on estimates for both the ADHD and non-ADHD groups. Other studies have reported considerably higher rates of critical incidents [43, 54], and it is likely that prison costs based on these would be considerably inflated compared with the estimates derived from the present data.

Our extrapolation method (using 3 months of data to estimate 1 year) may be limited in its accuracy. We used a one-year horizon for our HRQoL and service use estimates, and more time than this would have conferred too much uncertainty. Future research should address measuring utilities over more time, thereby providing a better foundation of QALY estimates beyond 1 year. Finally, the opportunity sampling method used may have introduced selection bias into the results, limiting their generalizability both within the prison and across other prisons.

## Conclusions

Research on HRQoL and costs related to adult ADHD is limited in the general population and is virtually non-existent in the prison population. We addressed this paucity of data on HRQoL, QALY, service utilization, and costs attributable to ADHD based on 1 year in prison. We performed HRQoL and cost analyses for adult prison inmates with ADHD based on a cross-section of the Scottish prison system in the UK.

Our study provides evidence that HRQoL of life is considerably poorer in adult male prison inmates with ADHD, with an adjusted reduction of 0.13 QALY. Affected health attributes extend beyond emotional and cognitive deficits, suggesting chronic effects of ADHD on health over the lifespan. ADHD may contribute to adverse health and quality of life directly through executive function and cognitive deficits; and co-morbid disorders. Combined costs within prison were significantly higher for those with ADHD and were driven by medical expenses. Service utilisation was for the most part limited to general practitioner services and nursing staff visits.

Approximately 80% of inmates considered to have ADHD did not receive a prior diagnosis — indicating that a significant proportion of adult prison inmates are inadequately identified and treated. This has policy implications for both the National Health and the prison service. There is a need for the prison service to develop improved awareness about ADHD in adult prisoners, including the clinical and behavoural presentation of ADHD. There is also a need to introduce a brief and reliable screen on admission, such as the 6-item B-BAARS, which has high sensitivity and specificity [25]. Furthermore, there is a need for the NHS to address the general absence of health service provision for adults with ADHD in prisons, as prisoners continue presenting multiple times for their health problems and seem to remain mis- or undiagnosed.

In 2015 the Ministry of Justice reported a population of 77,472 adult male inmates in the UK. Given the prevalence rate of 25.5% of ADHD among prisoners [55] and our estimated annual total cost per adult inmate with ADHD of £590, we estimate a total cost for medical and behaviour-related prison care of approximately £11.7 million per year. This cost estimate, however, is conservative as it is seemingly driven by general medical expenses and not by critical incidents. There may be variability in the reporting of critical incidents in prisons, and prison care costs associated with behavioural disturbances may be much higher in other establishments.

ADHD is a prevalent mental health disorder, and a known risk factor for a series of adverse health and social outcomes. Population studies report the community (and society at large) bears considerable medical costs associated with ADHD [22, 23]. Although ADHD is disproportionately prevalent in prison, it is understudied and inadequately addressed in this context [55, 56].

Our results provide evidence that adult prisoners with ADHD represent a unique population with unmet needs and high costs. Given the Swedish study of patients showing a 32% reduction in criminality for men and 41% for women during periods when they were receiving ADHD medication [57], effective identification and treatment of ADHD may have important cost implications.

We recommend directing efforts to increase access to effective interventions for adult inmates with ADHD. Setting up provisions for better access to early diagnosis and treatment is likely to improve inmates’ HRQoL and decrease impairment related to ADHD symptoms and associated co-morbidities.

## Abbreviations

ADHD: Attention-deficit hyperactivity disorder
BSI: Brief Symptom Inventory
CJS: Criminal justice system
CPI: Consumer price index
DIVA-2: Diagnostic interview for ADHD in adults 2.0
DSM-5: 5th edition of Diagnostic and Statistical Manual of Mental Disorders
GLM: Generalised linear models
HRQoL: Health-related quality of life
HUI3: Health Utilities Index Mark 3
NHS: National Health Service
QALY: Quality-adjusted life years
